# Optimal annual body mass index change for preventing spontaneous preterm birth in a subsequent pregnancy

**DOI:** 10.1038/s41598-022-22495-4

**Published:** 2022-10-19

**Authors:** Sho Tano, Tomomi Kotani, Takafumi Ushida, Masato Yoshihara, Kenji Imai, Tomoko Nakano-Kobayashi, Yoshinori Moriyama, Yukako Iitani, Fumie Kinoshita, Shigeru Yoshida, Mamoru Yamashita, Yasuyuki Kishigami, Hidenori Oguchi, Hiroaki Kajiyama

**Affiliations:** 1 Department of Obstetrics and Gynecology, Nagoya University Graduate School of Medicine, Nagoya, Aichi Japan; 2 Department of Obstetrics, Perinatal Medical Center, Toyota Memorial Hospital, Toyota, Aichi Japan; 3 Division of Perinatology, Center for Maternal-Neonatal Care, Nagoya University Hospital, Nagoya, Aichi 466-8560 Japan; 4 Department of Obstetrics and Gynecology, Fujita Health University School of Medicine, Toyoake, Aichi Japan; 5 Data Science Division, Data Coordinating Center, Department of Advanced Medicine, Nagoya University Hospital, Nagoya, Aichi Japan; 6 Kishokai Medical Corporation, Nagoya, Aichi Japan

**Keywords:** Health care, Medical research, Risk factors

## Abstract

Preterm birth (PTB) is a leading cause of neonatal morbidity and mortality. Although PTB is known to recur, interpregnancy preventive strategies for PTB have not been established to date. Annual BMI change can serve as a specific target value for preventing obstetric complications during interpregnancy care/counseling. This value can also account for age-related weight gain (0.2 kg/m^2^/year). In a multicenter retrospective study, we investigated the optimal annual BMI change for preventing PTB recurrence using the data of individuals who had two singleton births from 2009 to 2019. The association between annual BMI change and spontaneous PTB (sPTB) was analyzed by separating cases of medically indicated PTB (mPTB) from those of sPTB. Previous history of sPTB was strongly associated with sPTB in the subsequent pregnancy (adjusted odds ratio [aOR], 12.7; 95% confidence interval [CI], 6.5–24.8). Increase in annual BMI was negatively associated with sPTB (aOR, 0.6; 95% CI 0.5–0.9). The sPTB recurrence rate was significantly lower in patients with an annual BMI change of ≥ 0.25 kg/m^2^/year than in those with an annual BMI change of < 0.25 kg/m^2^/year (7.7% vs. 35.0%, *p* = 0.011). Our findings suggest that age-related annual BMI gain between pregnancies may help prevent sPTB recurrence.

## Introduction

Preterm birth (PTB), defined by the World Health Organization (WHO) as the birth prior to 37 completed weeks of gestation, is a leading cause of infant morbidity and mortality and is a major issue worldwide^1,2^. PTB has two distinct clinical subtypes: spontaneous PTB (sPTB, onset of labor before or after preterm premature rupture of the fetal membrane at preterm gestations) and medically indicated PTB (mPTB, provider-initiated birth owing to maternal or fetal indications at preterm gestations)^3–5^. Both subtypes of PTB, which often require intensive neonatal care, and lead to moderate-to-severe handicaps in survivors, including cerebral palsy, neurodevelopmental delays, and hearing/visual impairment, remain a critical problem^6–10^. The PTB rate in some countries, such as the United States of America, has slightly fallen^11^, but globally, the PTB rate is rising, with a prevalence of > 10%^2,12^. WHO identified PTB as a "top ten" research priority for 2025^13^, resulting in an increase in the number of studies on the risk factors of PTB^14–16^ and measures for PTB prevention^17–19^.

Cervical cerclage and progesterone administration are currently considered management options for pregnant patients who are at high risk for sPTB; however, both approaches have shown limited and debatable effectiveness^17,20^. Additionally, no previous study has established a preventive strategy that can be employed during the interpregnancy period. Evidence of the efficacy of interpregnancy care/counseling is accumulating, and interpregnancy care/counseling has been reported to modify the risks of unfavorable outcomes in the subsequent pregnancy^21–23^.

The most significant risk factor for PTB is a history of PTB^5,24–27^. Thus, practical approaches for decreasing the prevalence of PTB are desirable in individuals with a history of PTB. Currently, it is recommended that such patients should avoid short intervals between pregnancies (< 6 months from birth to the next conception)^22^. However, there are no clear recommendations for weight management^28^, although interpregnancy body mass index (BMI) changes (BMI gain of ≥ 4 kg/m^2^ and BMI loss of > 2 kg/m^2^) have been shown to be a risk factor for PTB in subsequent pregnancies^3^. These weight values are equivalent to approximately 10 kg and 5 kg, respectively, in Japanese women of average height (157.9 cm). However, the use of these values in interpregnancy care/counseling may be inappropriate because they lack a timeframe, which makes the goal unclear.

We recently reported that ascertaining “annual BMI change” would be helpful in interpregnancy care/counseling for each risk-stratified woman, to prevent hypertensive disorders of pregnancy (HDP)^29^ and gestational diabetes mellitus (GDM) in the subsequent pregnancy^30^. Importantly, women of reproductive age also show age-related weight gain (natural gain, approximately 0.5 kg/year)^31–33^, but annual BMI change can also account for age-related BMI change.

Annual BMI change has been reported in many medical fields, including the fields of diabetes mellitus^34,35^, cancer^36,37^, obstructive sleep apnea^38^, and cardiovascular disease^39^. However, no study has focused on the association between annual BMI change and PTB. The risk factor profiles for sPTB and mPTB are different^3,5,16,40^; a part of mPTB overlaps with the HDP population, as reported previously^4,40,41^. Thus, this study aimed to evaluate whether annual BMI change during the interpregnancy period was associated with sPTB in the subsequent pregnancy.

## Results

### Participants

**Figure 1:**
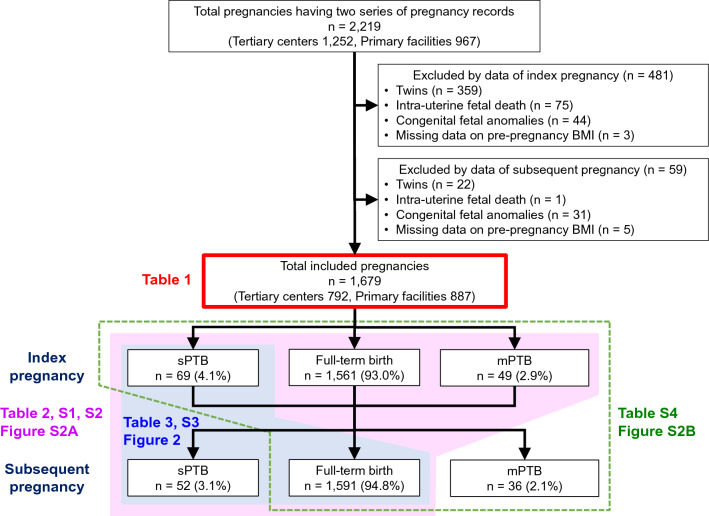
Flow chart of the study subjects. Clinical data were obtained from 2,219 individuals who gave birth twice at tertiary centers (n = 2) or primary maternity care units (n = 12). A total of 1679 individuals were eligible for this study, after the exclusion of 481 and 59 individuals based on their index and subsequent pregnancy data, respectively. BMI, body mass index; sPTB, spontaneous preterm birth; mPTB, medically indicated preterm birth.

A total of 2219 pregnant individuals (tertiary centers, n = 1252; primary maternity care units, n = 967) were included in this study. Among them, 540 were excluded because of twin births (n = 381), intrauterine fetal death (n = 76), congenital fetal anomalies (n = 75), and missing data for pre-pregnancy BMI (n = 8) during the index and subsequent pregnancies (Fig. 1). None of the patients showed cervical incompetence. The remaining 1679 pregnant individuals (tertiary centers, n = 792; primary maternity care units, n = 887) were eventually included in the study. The mean annual BMI change in the total population was 0.21 ± 0.83 kg/m^2^/year (data not shown).

### Comparison of clinical parameters between sPTB and full-term birth groups

sPTB occurred in 69/1679 (4.1%) index pregnancies and 52/1679 (3.1%) subsequent pregnancies (Fig. 1, Table 1). Regarding pregnancy characteristics during the index pregnancy (Table 1), patients with pre-pregnancy BMI < 18.5 kg/m^2^ were more prevalent in the sPTB group than in the full-term birth group (30.8% vs. 19.2%, *p* = 0.038), and sPTB was more prevalent in the sPTB group than in the full-term birth group (30.8% vs. 3.1%, *p* < 0.001).

**Table 1:** Baseline characteristics and perinatal outcomes.

	PTB in the subsequent pregnancy		Full-term birth in the subsequent pregnancy	p-value^a^	p-value^b^
	sPTB	mPTB			
	n = 52	n = 36	n = 1591		
**Index pregnancy**					
Tertiary center	37 (71.2)	34 (94.4)	721 (45.3)	< 0.001*	< 0.001*
Maternal age, years	30.4 ± 5.6	30.5 ± 5.1	30.5 ± 4.8	0.890	0.981
Maternal age ≥ 35 years	13 (25.0)	8 (22.2)	292 (18.4)	0.225	0.554
Maternal age < 20 years	2 (3.8)	0 (0)	27 (1.7)	0.233	1.000
Pre-pregnancy BMI, kg/m^2^	20.4 ± 3.3	22.1 ± 5.1	20.9 ± 3.4	0.308	0.192
Pre-pregnancy BMI ≥ 25.0 kg/m^2^	4 (7.7)	8 (22.2)	140 (8.8)	0.781	0.013*
Pre-pregnancy BMI < 18.5 kg/m^2^	16 (30.8)	7 (19.4)	305 (19.2)	0.038*	0.967
Smokers	1 (1.9)	0 (0.0)	16 (1.0)	0.284	1.000
Chronic hypertension	2 (3.8)	3 (8.3)	16 (1.0)	0.117	0.009*
Pre-exisiting DM	0 (0.0)	4 (11.1)	13 (0.8)	1.000	< 0.001*
Hyperthyroidism	0 (0.0)	1 (2.8)	12 (0.8)	1.000	0.323
Hypothyroidism	1 (1.9)	2 (5.6)	30 (1.9)	1.000	0.241
Nulliparity	38 (73.1)	26 (72.2)	1275 (80.1)	0.211	0.241
ART	5 (9.6)	4 (11.1)	133 (8.4)	0.593	0.519
Gestational body weight gain, kg	9.9 ± 4.0	9.4 ± 5.2	11.0 ± 3.9	0.051	0.019*
HDP	8 (15.4)	11 (30.6)	186 (11.7)	0.425	0.002*
PE	1 (1.9)	7 (19.4)	46 (2.9)	1.000	< 0.001*
GDM	5 (9.6)	3 (8.3)	67 (4.2)	0.083	0.211
GA at delivery, weeks	37.5 ± 2.4	36.9 ± 3.3	39.3 ± 1.9	< 0.001*	< 0.001*
Preterm birth (< 37 weeks)	17 (32.7)	12 (33.3)	89 (5.6)	< 0.001*	< 0.001*
Spontaneous	16 (30.8)	3 (8.3)	50 (3.1)	< 0.001*	0.110
Medically indicated	1 (1.9)	9 (25.0)	39 (2.5)	1.000	< 0.001*
Cesarean section	3 (5.8)	25 (69.4)	382 (24.0)	0.002*	< 0.001*
Neonatal sex, male	34 (65.4)	21 (58.3)	852 (53.6)	0.092	0.569
Neonatal height, cm	48.7 ± 2.7	46.4 ± 5.7	49.6 ± 2.6	0.015*	0.002*
Birthweight, g	2,706 ± 517	2,526 ± 743	3,002 ± 467	< 0.001*	< 0.001*
Large for gestational age infant	3 (5.8)	4 (11.1)	181 (11.4)	0.207	1.000
Small for gestational age infant	0 (0)	2 (5.6)	30 (1.9)	1.000	0.156
Placental weight, g	539.1 ± 106.7	493.4 ± 149.9	575.2 ± 111.0	0.021*	0.002*
**Interpregnancy**					
Pregnancy interval, years	2.3 ± 1.0	2.7 ± 1.7	2.2 ± 0.9	0.427	0.111
ΔBMI, kg/m^2^	0.04 ± 1.91	0.64 ± 2.09	0.44 ± 1.39	0.044*	0.580
Anuual BMI change, kg/m^2^/year	− 0.08 ± 1.41	0.31 ± 1.34	0.22 ± 0.78	0.007*	0.690
**Subsequent pregnancy**					
Maternal age, years	32.8 ± 5.7	33.2 ± 5.0	32.8 ± 5.0	0.994	0.570
Pre-pregnancy BMI, kg/m^2^	20.5 ± 3.3	22.7 ± 5.4	21.4 ± 3.6	0.079	0.144
Pre-pregnancy BMI ≥ 25.0 kg/m^2^	4 (7.7)	8 (22.2)	183 (11.5)	0.395	0.062
Pre-pregnancy BMI < 18.5 kg/m^2^	14 (26.9)	7 (19.4)	270 (17.0)	0.062	0.696
ART	5 (9.6)	3 (8.3)	132 (8.3)	0.616	0.755
Gestational body weight gain, kg	8.4 ± 3.6	7.4 ± 4.8	10.3 ± 3.6	< 0.001*	0.019*
HDP	1 (1.9)	14 (38.9)	122 (7.7)	0.176	< 0.001*
PE	0 (0.0)	12 (33.3)	19 (1.2)	1.000	< 0.001*
GDM	4 (7.7)	7 (19.4)	142 (8.9)	1.000	0.031*
GA at delivery, weeks	35.4 ± 1.7	33.9 ± 3	39.2 ± 1.1	< 0.001*	< 0.001*
22 to < 32 weeks	4 (7.7)	5 (13.9)	0 (0.0)	< 0.001*	< 0.001*
32 to < 37 weeks	48 (92.3)	31 (86.1)	0 (0.0)	< 0.001*	< 0.001*
Cesarean section	0 (0.0)	32 (88.9)	374 (23.5)	< 0.001*	< 0.001*
Neonatal sex, male	29 (55.8)	16 (44.4)	807 (51.5)	0.541	0.405
Neonatal height, cm	46.6 ± 2.8	43.5 ± 6.1	50.0 ± 1.7	< 0.001*	< 0.001*
Birthweight, g	2432 ± 424	1977 ± 780	3095 ± 367	< 0.001*	< 0.001*
Large for gestational age infant	7 (13.5)	1 (2.8)	183 (11.5)	0.756	0.345
Small for gestational age infant	0 (0.0)	4 (18.2)	12 (0.9)	1.000	< 0.001*
Placental weight, g	543.2 ± 109.9	450.7 ± 200.4	587.6 ± 108.1	0.004*	< 0.001*

PTB, preterm birth; sPTB, spontaneous PTB; mPTB, medically indicated PTB; GDM, gestational diabetes mellitus; BMI, body mass index; DM, diabetes mellitus; ART, assisted reproductive technology; GA, gestational age; HDP, hypertensive disorders of pregnancy; PE, preeclampsia.

Data are presented as means ± standard deviation for continuous variables and n (%) for discrete variables.

*Statistically significant.

Significant values are in bold.

^a^p-value for compareing full-term birth group and sPTB; ^b^p-value for compareing full-term birth group and mPTB.

Among the interpregnancy variables, ΔBMI and annual BMI change were lower in the sPTB group were lower than those in the full-term birth group (0.04 ± 1.91 kg/m^2^ vs. 0.44 ± 1.39 kg/m^2^, *p* = 0.044; − 0.08 ± 1.41 kg/m^2^/year vs. 0.22 ± 0.78 kg/m^2^/year, *p* = 0.007, respectively, Table 1).

### Risk factors for sPTB in the subsequent pregnancy

According to multivariable regression analysis using classified annual BMI change with using annual BMI change of ≥ 0 to < 0.14 kg/m^2^/year as a reference, as shown in Fig. S2A, sPTB less frequently occurred in the subsequent pregnancy when there was increment in annual BMI change: the aOR of aPTB for annual BMI change of ≥ 0.72 kg/m^2^/year was 0.3 (95%CI 0.1–1.0). The group with minimal weight gain (≥ 0 to < 0.14 kg/m^2^/year), which was used as a reference, had a similar frequency of sPTB as the group with weight loss (≥ − 0.3 to < 0, and < − 0.3 kg/m^2^/year).

In a multivariable analysis for predicting sPTB in the subsequent pregnancy (Table 2), having a history of sPTB in the index pregnancy and lower annual BMI change were independently associated with sPTB in the subsequent pregnancy. The odds ratio of sPTB was 0.7 per 1.0 kg/m^2^/year increment of annual BMI change. After adjustments for maternal age, underweight status, nulliparity, sPTB in the index pregnancy, and pregnancy interval, the odds ratio became even lower (0.6 per 1.0 kg/m^2^/year). Multivariable regression analyses using other possible confounders were also conducted as sensitivity analyses, and similar results were obtained (Table S1).

**Table 2:** Univariable and multivariable logistic regression analysis of variables potentially associated with sPTB in the subsequent pregnancy (n = 1643).

		Spontaneous PTB in the subsequent pregnancy				
		n/N (%)	cOR	95%CI	aOR	95%CI
Index pregnancy	Maternal age ≥ 35 years	13/305 (4.3)	1.5	(0.8–2.8)	1.4	(0.7–2.7)
	Maternal age < 20 years	2/29 (6.9)	2.3	(0.5–10.0)	1.6	(0.3–9.2)
	Pre-pregnancy BMI < 18.5 kg/m^2^	16/321 (5.0)	1.9*	(1.0–3.4)	1.7	(0.9–3.3)
	Nulliparity	38/1313 (2.9)	0.7	(0.4–1.3)	0.7	(0.4–1.4)
	Spontaneous PTB	16/66 (24.2)	13.7*	(7.1–26.3)	12.7*	(6.5–24.8)
Inter-pregnancy	Pregnancy interval, years	–	1.1	(0.9–1.5)	1.1	(0.8–1.5)
	Anuual BMI change, kg/m^2^/year	–	0.7*	(0.5–0.9)	0.6*	(0.5–0.9)

n/N: The number of sPTB events in the subsequent pregnancy/the number of patients for each variables; cOR, clude odds ratio; aOR, adjusted OR; CI, confidence interval; BMI, body mass index; PTB, preterm birth.

*Statistically significant.

We further analyzed the association between BMI change and sPTB in the subsequent pregnancy by stratifying the patients according to whether or not they were underweight (pre-pregnancy BMI < 18.5 kg/m^2^) in the index pregnancy (Table S2). For non-underweight individuals, increasing annual BMI change had a negative association with sPTB in the subsequent pregnancy (aOR 0.6, 95%CI 0.4–0.9), but for underweight individuals, pregnancy interval (aOR 1.7, 95%CI 1.1–2.9), rather than annual BMI change (aOR 1.3, 95%CI 0.5–3.6), was significantly associated with sPTB in the subsequent pregnancy after adjustment for maternal age, nulliparity, sPTB in the index pregnancy, and annual BMI change.

### Association between annual BMI change and sPTB recurrence

**Figure 2:**
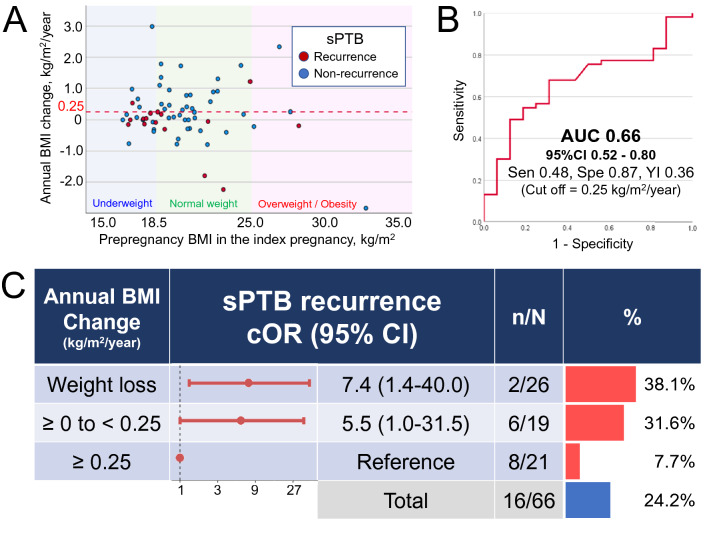
Association between annual BMI change during the interpregnancy interval and recurrent sPTB among individuals with a history of sPTB. (**A**) Pre-pregnancy BMI in the index pregnancy and annual BMI change. Red and blue dots indicate values in the sPTB recurrence group and non-recurrence group, respectively. Red dash line indicates an annual BMI change of 0.25 kg/m^2^/year. (**B**) Receiver operating characteristic curve of the annual BMI change for the development of sPTB recurrence in the subsequent pregnancy. (**C**) Crude odds ratios for recurrent sPTB with 95% CI in each category of annual BMI change, with an annual BMI change of ≥ 0.25 kg/m^2^/year as the reference. The right-sided graph shows the sPTB recurrence in each category of annual BMI change. The number of recurrent sPTBs/total number is also shown as n/N. BMI, body mass index; AUC, area under the curve; Sen, sensitivity; Spe, specificity; YI, Youden index; sPTB, spontaneous preterm birth; cOR, crude odds ratio; CI confidence interval.

We analyzed the data of individuals who experienced sPTB in the index pregnancy. The scatter plot (Fig. 2A) shows the relationship between pre-pregnancy BMI in the index pregnancy and annual BMI change during the interpregnancy period. Annual BMI change was significantly lower in the sPTB recurrence group (red dots) than in the non-recurrence group (blue dot) (analysis of covariance [ANCOVA], adjusted for pre-pregnancy BMI in the index pregnancy, *p* = 0.038). The area under the receiver operating characteristic curve for annual BMI change among patients who experienced sPTB in the index pregnancy for sPTB recurrence in the subsequent pregnancy was 0.66 (95% CI 0.52–0.80: Fig. 2B). According to the maximum Youden index (Table S3), the optimal cutoff value of annual BMI change was 0.25 kg/m^2^/year, with a sensitivity of 48% and specificity of 94%. sPTB recurrence in individuals with an annual BMI change of ≥ 0.25 kg/m^2^/year was significantly lower than that in those with an annual BMI change of < 0.25 kg/m^2^/year (2/26 [7.7%] vs. 14/40 [35.0%], *p* = 0.011, data not shown). A similar increasing trend of the crude odds ratio (cOR) was observed between annual BMI change ≥ 0.0 to < 0.25 kg/m^2^/year and weight loss (< 0.0 kg/m^2^/year) groups, and the corresponding cORs were 5.5 and 7.4, respectively, with annual BMI change of ≥ 0.25 kg/m^2^/year group serving as the reference (Fig. 2C).

We further analyzed the association between annual BMI change and sPTB recurrence by adding gestational weight gain during the subsequent pregnancy as a variable (Table 3). The OR of sPTB recurrence was 0.5 per 1.0 kg/m^2^/year increment of annual BMI change. After adjustments for maternal age and underweight status in the index pregnancy, pregnancy interval, and gestational weight gain in the subsequent pregnancy, the odds ratio became lower (0.4 per 1.0 kg/m^2^/year). However, gestational weight gain during the subsequent pregnancy was not statistically significant (aOR 0.9, 95%CI 0.7–1.1).

**Table 3:** Univariable and multivariable logistic regression analysis of variables potentially associated with sPTB recurrence (n = 66).

		sPTB recurrence	Non-recurrence	p-value	sPTB recurrence			
					cOR	95%CI	aOR	95%CI
		n = 16	n = 50					
Index-pregnancy	Maternal age ≥ 35 years	5 (31.3)	11 (22.0)	0.509	3.3	(0.2–55.4)	1.7	(0.3–8.3)
	Maternal age < 20 years	1 (6.3)	1 (2.0)	0.429	1.6	(0.5–5.6)	2.1	(0.1–41.1)
	Pre-pregnancy BMI < 18.5 kg/m^2^	7 (43.8)	13 (26.0)	0.217	2.2	(0.7–7.2)	1.9	(0.5–8.1)
Inter-pregnancy	Pregnancy interval, years	2.8 ± 1.5	2.1 ± 0.7	0.060	2.3*	(1.1–4.6)	2.2	(1.0–4.8)
	Anuual BMI change, kg/m^2^/year	− 0.15 ± 0.81	0.34 ± 0.91	0.056	0.5	(0.3–1.1)	0.4*	(0.2–1.0)
Subsequent-pregnancy	Gestational weight gain, kg	8.1 ± 3.5	9.6 ± 3.3	0.131	0.9	(0.7–1.1)	0.9	(0.7–1.1)

cOR, clude odds ratio; aOR, adjusted OR; CI, confidence interval; BMI, body mass index; sPTB, spontaneous preterm birth.

*Statistically significant.

### Analyses of mPTB in the subsequent pregnancy after excluding sPTB in the subsequent pregnancy

mPTB occurred in 49/1679 (2.9%) index pregnancies and 36/1679 (2.1%) subsequent pregnancies (Fig. 1, Table 1). Regarding index pregnancy characteristics (Table 1), the mPTB group showed a significantly higher prevalence of individuals with pre-pregnancy BMI ≥ 25.0 kg/m^2^ (22.2% vs. 8.8%, *p* = 0.013), chronic hypertension (8.3% vs. 1.0%, *p* = 0.009), pre-exisiting diabetes mellitus (DM; 11.1% vs. 0.8%, *p* < 0.001), HDP (30.6% vs. 11.7%, *p* = 0.002), preeclampsia (PE; 19.4% vs. 2.9%, *p* < 0.001), and mPTB (25.0% vs. 2.5%, *p* < 0.001), than the full-term birth group. Among interpregnancy variables, patients with mPTB had slightly longer interpregnancy intervals (2.7 ± 1.7 years vs. 2.2 ± 0.9 years, *p* = 0.111) and larger annual BMI change (0.31 ± 1.34 kg/m^2^/year vs. 0.22 ± 0.78 kg/m^2^/year, *p* = 0.690), but neither were not statistically significant.

The shape of the association between annual BMI change and mPTB in the subsequent pregnancy was also examined by classifying the individuals into six annual BMI change groups. After considering confounding factors, including pre-pregnancy BMI ≥ 25.0 kg/m^2^, the mPTB rate in each group was curvilinear (U-shaped), although this was not statistically significant (Fig. S2B).

To determine the risk of mPTB in the subsequent pregnancy, the following variables were extracted as significantly associated factors according to the multivariable regression analyses (Table S4): chronic hypertension (3/19 [15.8%]; aOR, 5.2; 95% CI 1.2–22.0), pre-exisiting DM (4/17 [23.5%]; aOR, 19.0; 95% CI 4.8–75.6), mPTB in the index pregnancy (9/48 [18.8%]; aOR, 11.0; 95% CI 4.4–27.4), and longer pregnancy interval (aOR, 1.5; 95% CI 1.1–1.9).

## Discussion

To our knowledge, this is the first study to demonstrate that the annual BMI change in the interpregnancy period was associated with sPTB in the subsequent pregnancy. Annual BMI change and a history of sPTB were independently associated with sPTB occurrence. Furthermore, maintenance of an annual BMI gain of ≥ 0.25 kg/m^2^/year was suggested to be a target value for preventing sPTB recurrence in individuals with a history of sPTB. The subsequent development of mPTB was significantly associated with chronic hypertension, pre-exisiting DM, a history of mPTB, and long pregnancy interval.

Several previous studies have suggested that maternal pre-pregnancy overweight/obesity was related to increased rates of both subtypes of PTB^42,43^, but sPTB alone was reported to be associated with obesity (BMI ≥ 30.0 kg/m^2^), whereas both overweight and obesity (BMI ≥ 25.0 kg/m^2^) were related to mPTB^3,42^. In this study setting, with a pre-pregnancy BMI cutoff value of ≥ 25.0 kg/m^2^ (overweight/obesity), an increasing trend of mPTB was observed in individuals with overweight/obesity, but overweight/obesity was not significantly associated with sPTB in the subsequent pregnancies. It might have resulted from that overweight/obesity was less prevalent in the present study population. Meanwhile, underweight is widely considered a risk factor for sPTB^44,45^, as was also observed in the present study. Additionally, patients at a high risk for sPTB and mPTB were shown to be those with a history of sPTB and mPTB, respectively, which is consistent with the results of previous studies^46–54^.

In the present study, for sPTB occurrence in the subsequent pregnancy, a negative association was observed between annual BMI change and sPTB in the subsequent pregnancy, consistent with previous reports that weight loss is considered a risk factor for sPTB^3,55^. However, in underweight individuals, a long pregnancy interval, rather than BMI change reduction, was associated with sPTB in the subsequent pregnancy. It seems possible that other risk factors may be strongly associated with sPTB in underweight individuals. Furthermore, sPTB recurrence was examined among the patients with a history of sPTB. As this was a retrospective study, further studies are needed to determine whether annual BMI change is a single predictor of sPTB recurrence, but the cutoff value for annual BMI change was, notably, 0.25 kg/m^2^/year. This value is very close to the mean annual BMI change in the present study, 0.21 kg/m^2^/year, consistent with previously reported data for age-related weight gain^29,31,32^. Although it is known that weight loss is a risk factor for sPTB^3,55^, the present study newly revealed that both weight gain below 0.25 kg/m^2^/year and weight loss are associated with sPTB recurrence. Age-related weight gain may be essential for preventing sPTB recurrence. Additionally, gestational weight gain in the subsequent pregnancy was not significantly associated with sPTB recurrence. The association between gestational weight gain and PTB remains undetermined^56^. Thus, the target for preventing sPTB recurrence is annual BMI gain during the interpregnancy period, rather than gestational weight gain.

In contrast to the findings on sPTB, a U-shaped association was detected between annual BMI change and mPTB in the subsequent pregnancy, but the present study failed to reveal the statistically significant association between annual BMI change and mPTB. HDP is a major cause of mPTB^4,40,41^. In the present study, 38.9% of mPTBs were also associated with HDP (14/36). Moreover, 38.7% of patients with preeclampsia (12/31), a severe type of HDP, had a preterm birth. The present study could not achieve the cutoff value of annual BMI gain for preventing mPTB, including preterm HDP, because of the small population of mPTB cases. However, an annual BMI gain of ≥ 0.6 kg/m^2^/year has been reported to be associated with HDP recurrence^29^. Therefore, this finding may be utilized to prevent mPTB in the subsequent pregnancy. These results suggest that it seems acceptable to consider individuals whose interpregnancy annual BMI gain of 0.25–0.6 kg/m^2^/year may be associated with a low rate of adverse events in the subsequent pregnancy.

## Strengths and limitations

This study had several strengths. First, to our knowledge, this is the first study to assess the association between PTB in the subsequent pregnancy and annual BMI changes during the interpregnancy period. Second, this multicenter study was based on data from both tertiary care centers and primary maternity care units, which enabled the inclusion of patients with various risk levels and minimized selection bias. Third, we independently examined factors associated with each of the two subtypes of PTB and analyzed the association between annual BMI change and PTB, stratified by a history of sPTB or mPTB. Thus, the findings of this study can be considered more informative than those of studies with unclassified PTB, because the pathologies of sPTB and mPTB are different from each other.

This study had several limitations. First, only individuals who gave birth twice were included. Individuals who developed infertility after the index pregnancy or those who had a miscarriage in a subsequent pregnancy were not included. However, the present study focused on PTB, and the effect of these exclusions on the results would have been minimal. Second, postpartum weight was not followed up. Annual BMI change was not measured over annual check-ups, rather it was calculated using the interpregnancy BMI change (ΔBMI) and the interpregnancy interval, as in previous studies^29,30^. However, the mean weight change from pre-pregnancy to one year after parturition, which is approximately 2 years, was reported to be 0.9 kg^57^, which was comparable to the age-related weight gain^31–33^. Since the mean pregnancy interval in the present study was 2.3 years, the difference between the actual and calculated BMI change is not expected to be significant. However, further prospective studies using actual measured values are required to validate the present data. Third, self-reported body weight data was used in this study. Nevertheless, as body weight is routinely assessed during antenatal care in hospitals and clinics during the first trimester, the difference between the self-reported and actual values might be minimal. Fourth, data regarding the prophylactic procedures, including cervical cerclage or progesterone administration, for individuals with a history of sPTB could not be included in the analyses. Cervical cerclage or progesterone administration are currently uncommon in Japan. Cervical cerclage is performed for patients with cervical incompetence, but no patient had cervical incompetence in this study. These factors seem to be unassociated with the annual BMI change, but future studies with larger populations are needed to confirm these findings. Finally, the values of annual BMI change were also affected by the low rates of PTB and overweight/obesity in this study population. Therefore, specific values should be determined in each country or race.

## Conclusion

In individuals with a history of sPTB who had relatively high odds ratios for sPTB in the subsequent pregnancy, an annual BMI change of < 0.25 kg/m^2^/year was significantly associated with recurrent sPTB. Thus, age-related weight gain may be beneficial for sPTB prevention. However, the findings do not clarify whether postpartum weight management can reduce the incidence of recurrent sPTB. We believe our study findings can serve as the basis for future research on this topic.

## Methods

### Study population

This multicenter retrospective study used electronic medical record data of pregnant individuals who gave birth at one of two tertiary centers in Aichi Prefecture (Nagoya University Hospital and TOYOTA Memorial Hospital) or at one of 12 private maternity facilities (Kishokai Medical Corporation located in Aichi and Gifu Prefectures) between 2009 and 2019^29^. Individuals aged ≥ 15 years whose medical records for both the index and the subsequent pregnancy were available were included in this study. We assessed the medical records directly and evaluated laboratory and clinical data, if necessary. The exclusion criteria were as follows: multiple pregnancies, intrauterine fetal death, congenital fetal anomalies (chromosomal anomalies and malformations), and missing data for pre-pregnancy BMI (Fig. 1).

### Definitions of the variables

We used self-reported maternal height and pre-pregnancy body weight to calculate BMI (kg/m^2^), which was categorized as < 18.5 kg/m^2^ (underweight), 18.5–24.9 kg/m^2^ (normal weight), and ≥ 25.0 kg/m^2^ (overweight/ obesity) according to WHO classifications^58^.

Pregnant individuals were divided into three groups according to the status of the subsequent pregnancy: sPTB, mPTB, and full-term birth (≥ 37 weeks of gestation). sPTB and mPTB were defined on the basis of a previous study^3^. sPTB was defined as childbirth after the onset of labor before or after preterm premature rupture of the fetal membrane, before 37 weeks of gestation, mPTB was defined as PTB via a Cesarean section or induction of labor, before the onset of labor, due to maternal or fetal indications, including preeclampsia, fetal growth restriction, and a non-reassuring fetal status, based on a review of medical records. Thus, cases of PTB without a Cesarean section or induction of labor were classified as sPTB, even if the patient had preeclampsia. Assisted reproductive technology was defined as conception after in vitro fertilization or intracytoplasmic sperm injection. Gestational weight gain was defined as the bodyweight change between the pre-pregnancy period and parturition. Patients with a fasting plasma glucose level of ≥ 126 mg/dL during pregnancy or a hemoglobin A1c (HbA1c) level of ≥ 48 mmol/mol (6.5%) were defined as having pre-exisiting DM. Chronic hypertension was defined as diastolic blood pressure ≥ 90 mmHg or systolic blood pressure ≥ 140 mmHg before 20 weeks of gestation. HDP was defined as hypertension after 20 weeks of gestation. Preeclampsia was defined using a simplified classification^59^: HDP in combination with proteinuria (positive proteinuria dipstick test [≥ 0.3 g/L], protein/creatinine ratio of ≥ 30 mg/mmol, or urine protein extraction of ≥ 300 mg/day) or fetal growth restriction. GDM was diagnosed using oral glucose tolerance test (75g-OGTT): fasting plasma glucose level ≥ 92 mg/dL or the 1-h and 2-h plasma glucose levels after 75g-glucose loading ≥ 180 mg/dL or ≥ 153 mg/dL, respectively. Determination of small and large for gestational age infants was based on the Japanese standards for birth weight, which is according to the pregnancy duration^60^. Gestational age was routinely estimated by the expected date of delivery, which was determined by the last menstruation cycle and the measurement of the crown-rump length or the age of the embryo and the date of transfer in assisted reproductive technology pregnancies. As shown in Fig. S1^29,30^, ΔBMI was defined according to previous studies^3,29,30^ as change in pre-pregnancy BMI from the index pregnancy to that of subsequent pregnancy. Pregnancy interval was defined as the interval from the expected date of delivery of the index pregnancy to that of the subsequent pregnancy, which is equivalent to the interval between two pregnancies^29,30^. Annual BMI change during the interpregnancy period was calculated as ΔBMI/pregnancy interval.

### Statistical analysis

As the present study was an exploratory study to compare sPTB and mPTB groups individually with the full-term birth group, the clinical characteristics and other parameters of patients who experienced sPTB/mPTB were separately compared with those of patients who experienced full-term birth in the subsequent pregnancy. The χ^2^ test or Fisher’s test was used for categorical variables, and Student’s *t*-test or Mann–Whitney *U* test was used for continuous variables. The cORs and aORs for sPTB in the subsequent pregnancy were calculated using univariable and multivariable logistic regression analyses, after excluding mPTB in the subsequent pregnancy. The values for mPTB in the subsequent pregnancy were calculated after excluding sPTB in the subsequent pregnancy. Variables used in the univariable and multivariable analyses were selected based on previous studies^3,5,24–27^. They included maternal age ≥ 35 years, maternal age < 20 years, pre-pregnancy BMI ≥ 25.0 kg/m^2^, pre-pregnancy BMI < 18.5 kg/m^2^, chronic hypertension, overt DM, HDP, GDM, gestational weight gain, the presence of sPTB/mPTB in the index pregnancy, pregnancy interval, and annual BMI change during the interpregnancy period. To examine the shape of the association between annual BMI change and sPTB/mPTB in the subsequent pregnancy, we classified annual BMI change into six groups according to the distribution and calculated each aOR of classified annual BMI change using 0–0.14 kg/m^2^/year as a reference. ANCOVA was conducted to examine differences in annual BMI change between the sPTB/mPTB recurrence group and the non-recurrence group, with pre-pregnancy BMI in the index pregnancy as a covariate. In addition, receiver operating characteristic analysis was performed to evaluate the association between annual BMI change and sPTB recurrence in the subsequent pregnancy. The best cutoff value was determined using the Youden index (sensitivity + specificity − 1). Statistical significance was set at a p-value of < 0.05. Statistical analyses were conducted using SPSS ver. 28.0 for Windows software (IBM, Chicago, IL, USA).

### Ethical approval

This study was approved by the Nagoya University Hospital ethics committee (approval number: 2015–0415) in accordance with the guidelines of the Declaration of Helsinki and STROBE guidelines. Furthermore, the ethics committee waived the requirement for written informed consent because of the retrospective nature of the study.

## Supplementary Information

Supplementary Legends.

Supplementary Figure S1.

Supplementary Figure S2.

Supplementary Tables.

## Data Availability

The data are available upon reasonable request and with the permission of Kishokai Medical Corporation.
