# Supplementary material for: Optimal annual body mass index change for preventing spontaneous preterm birth in a subsequent pregnancy
Source: Sci Rep. 2022 Oct 19;12:17502. doi: 10.1038/s41598-022-22495-4 (PMC9582014; doi:10.1038/s41598-022-22495-4)
Supplement: Supplementary file 1 — Supplementary Legends. [file 41598_2022_22495_MOESM1_ESM.docx]

**Figure S1. Overview of the definitions of terms.**

We modified the figure in the previous report [29, 30]. Overall BMI change (ΔBMI) was defined as the change in pre-pregnancy BMI from the index pregnancy to the subsequent pregnancy. Pregnancy interval was defined as the interval from the expected date of delivery of the index pregnancy to that of the subsequent pregnancy, which is equivalent to the interval between the two conceptions. Therefore, annual BMI change was calculated as ΔBMI per pregnancy interval. Gestational weight gain was defined as the weight difference between the pre-pregnancy body weight and that before parturition.

BMI, body mass index.

**Figure S2. Association between annual BMI change during the interpregnancy interval and PTB**

Adjusted odds ratios for spontaneous preterm birth (sPTB, A) and medically indicated PTB (mPTB, B) in the subsequent pregnancy with 95% CI in each category of annual BMI change, with an annual BMI change of ≥0 to <0.14 kg/m2/year as the reference. A. The multivariable models were adjusted for maternal age <20 years, maternal age ≥35 years, pre-pregnancy BMI <18.5 kg/m2, nulliparity, sPTB in the index pregnancy, and pregnancy interval. The right-sided graph shows the rate of sPTB in each category of annual BMI change. The number of sPTBs/total number is also shown as n/N. B. The multivariable models were adjusted for maternal age ≥35 years, pre-pregnancy BMI ≥25.0 kg/m2, nulliparity, mPTB in the index pregnancy, and pregnancy interval. The right-sided graph shows the rate of mPTB in each category of annual BMI change. The number of mPTBs/total number is also shown as n/N.

BMI, body mass index; aOR, adjusted odds ratio; CI, confidence interval.

**References**

29. Tano, S., et al., Annual body mass index gain and risk of hypertensive disorders of pregnancy in a subsequent pregnancy. Sci Rep, 2021. 11(1): p. 22519.

30. Tano S., et al., Annual body mass index gain and risk of gestational diabetes mellitus in a subsequent pregnancy. Frontiers in Endocrinology, in press.
