# Supplementary figures and images for: Optimal annual body mass index change for preventing spontaneous preterm birth in a subsequent pregnancy
Source: Sci Rep. 2022 Oct 19;12:17502. doi: 10.1038/s41598-022-22495-4 (PMC9582014; doi:10.1038/s41598-022-22495-4)

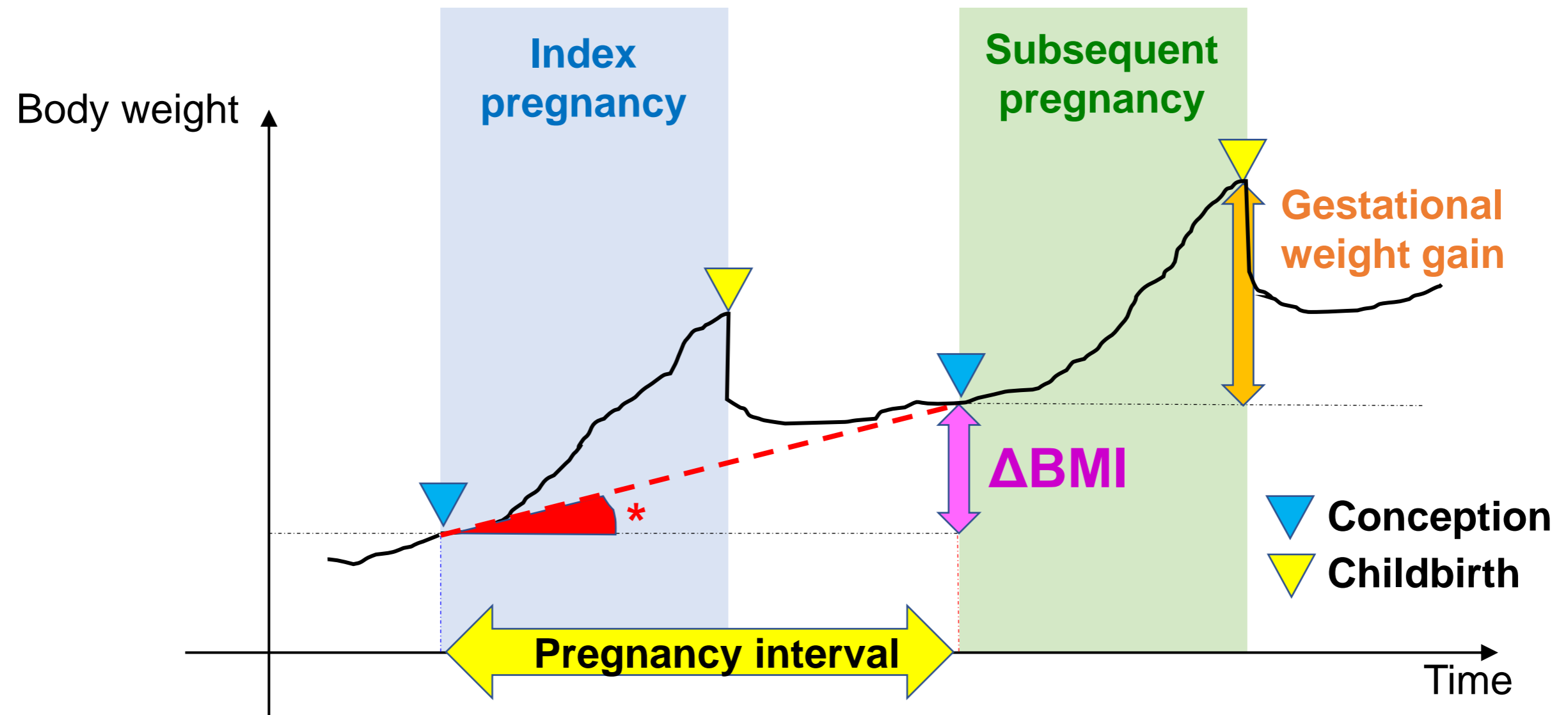

**\*Annual BMI change =  $\Delta\text{BMI} / \text{Pregnancy interval}$  (kg/m<sup>2</sup>/year)**

Supplement: Supplementary file 2 — Supplementary Figure S1. [file 41598_2022_22495_MOESM2_ESM.pdf]

A

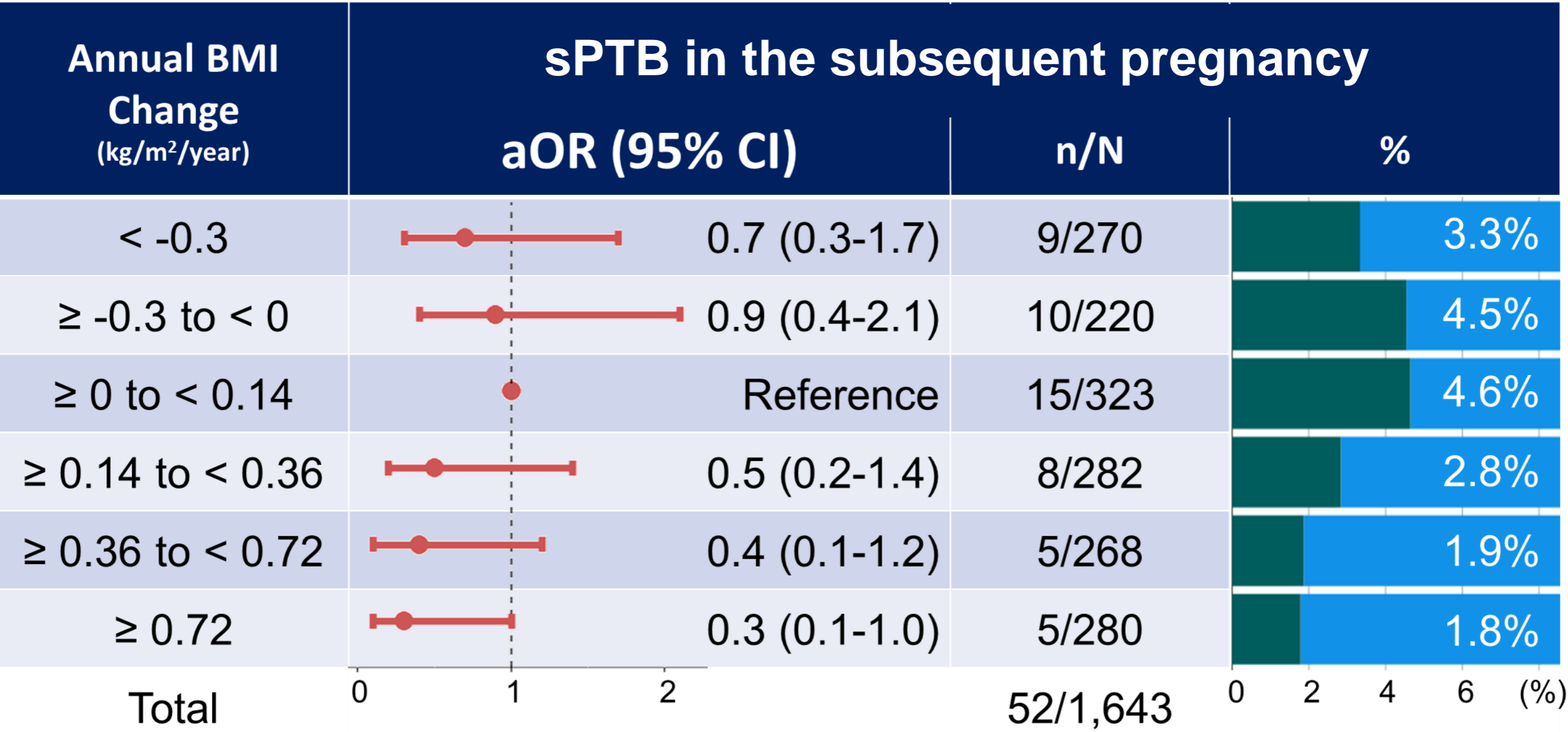

B

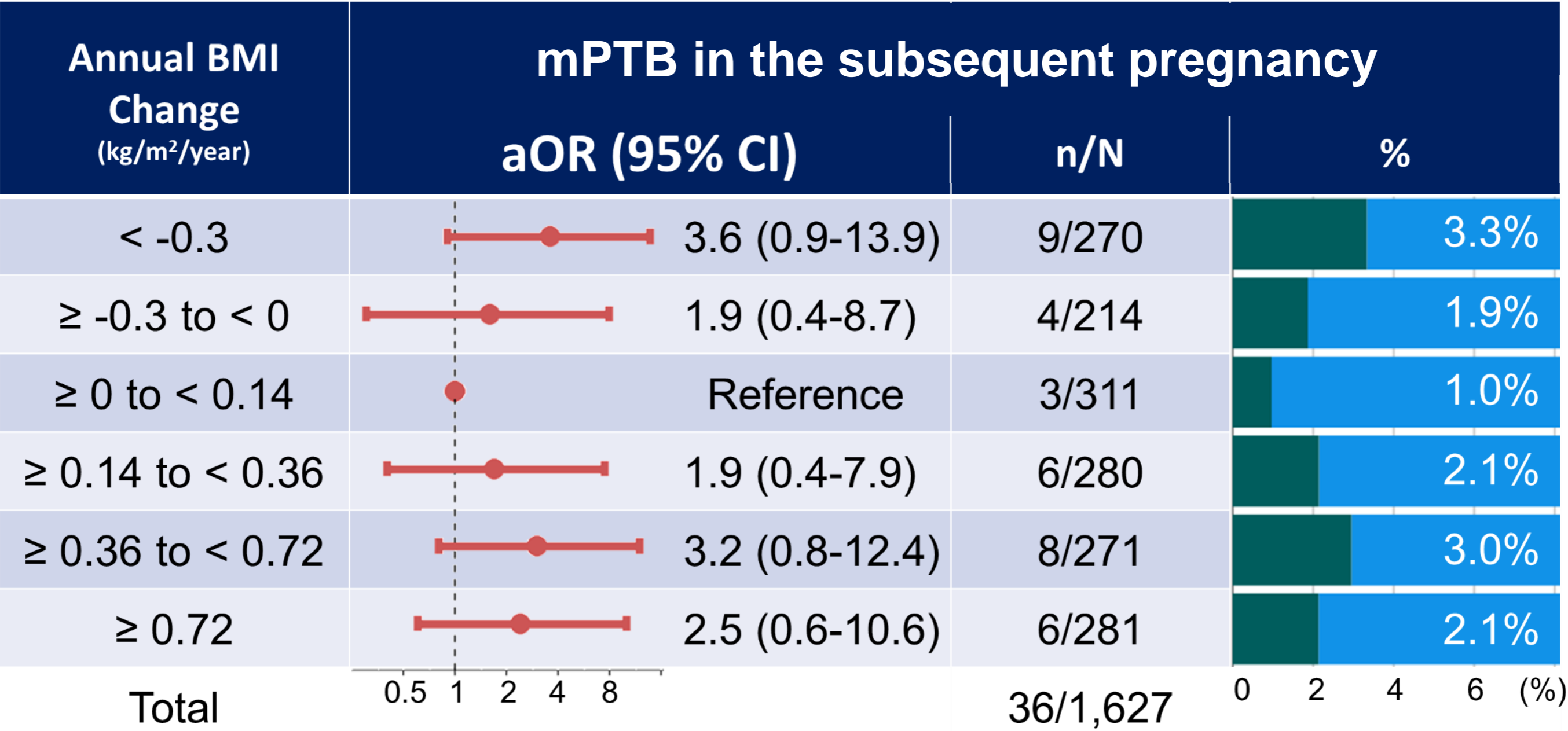

Supplement: Supplementary file 3 — Supplementary Figure S2. [file 41598_2022_22495_MOESM3_ESM.pdf]
